# Vocal efficiency in crows

**DOI:** 10.1007/s10071-025-01985-8

**Published:** 2025-07-31

**Authors:** Claudia A. F. Wascher, Mason Youngblood

**Affiliations:** 1 Behavioural Ecology Research Group, School of Life Sciences, Anglia Ruskin University, Cambridge, UK; 2 Institute for Advanced Computational Science, Stony Brook University, Stony Brook, NY USA

**Keywords:** Carrion crows, Communicative efficiency, Hooded crows, Hybrids, Linguistic laws, Vocal communication

## Abstract

Many communicative systems have been selected for efficiency, shaped by the trade-off between information transmission and energetic or temporal constraints. Linguistic laws such as Menzerath’s law—predicting shorter elements in longer sequences—have emerged as widespread principles across vocal communication in many species. While these laws have been predominantly studied at the species level, the influence of individual and social factors remain underexplored. In this study, we investigated adherence to Menzerath’s law in the vocal communication of carrion crows, *Corvus corone corone*, hooded crows, *Corvus corone cornix* and hybrids. Our findings show that crow call sequences adhere to Menzerath’s law, with shorter calls occurring in longer sequences, demonstrating structural efficiency in vocal communication. In carrion crows specifically, we analysed call sequences in relation to individual characteristics (sex, age) and social variables (group size, dominance status, strength of affiliative relationships). Interestingly, adherence to Menzerath’s law was stronger in females and younger individuals, while no effects were found for group size, dominance, or affiliative relationships. This study provides the first evidence of Menzerath’s law in corvid vocal communication and suggests that individual-level traits, rather than broader social dynamics, may shape vocal efficiency. These findings broaden our understanding of widespread principles in animal communication and raise new questions about the ontogeny and flexibility of vocal efficiency in complex social species.

**Supplementary Information:**

The online version contains supplementary material available at 10.1007/s10071-025-01985-8.

## Introduction

Biological systems have evolved to operate efficiently under the constraints of energy and time, ensuring survival and reproductive success (Igamberdiev 2023). This is also the case for a wide range of communication systems, such as human language (Gibson et al. 2019) and vocal communication in non-human animals (Endler 1993; Semple et al. 2022). Communication systems typically evolve to convey critical information accurately, which often requires an increase of signal complexity. For example in the context of mating, increased signal complexity is associated with increased mating success (Choi et al. 2022; Pollard and Blumstein 2012). In an anti-predator context, complex signals can convey information such as predator type and level of risk (Suzuki 2014) and social factors such as increased group sizes, and cohesiveness of social bonds requires increasingly complex communication systems such as increases in vocal repertoires or diversity of signals (Peckre et al. 2019). The need for complexity for information transmission needs to be balanced with time and energy constraints, consequently selecting communication systems for efficiency and allowing to optimize the benefits resulting from information transmission while reducing the costs of signal production (Endler 1993).

One widespread way to reduce costs of communicating an increase signalling efficiency is the reduction of communication time, for example by minimizing the length of a signal. In the context of human language, this efficiency is traditionally quantified as linguistic laws, such as Zipf’s law of brevity, which predicts a negative relationship between the length of words and how often they are used (Zipf 1949) and Menzerath’s law, which predicts that longer sequences will be comprised of shorter items to balance production costs (Menzerath 1954). In recent years, empirical evidence is accumulating suggesting that these information theoretical principles are widespread across a wide range of taxa and communication modalities. Menzerath’s law is common for example in bird song (Favaro et al. 2020; James et al. 2021; Lewis et al. 2023; Youngblood 2024), primates (Gustison et al. 2016; Huang et al. 2020; Valente et al. 2021), bats (Zhang et al. 2024), or whale vocalisations (Youngblood 2025). Adherence to Menzerath’s law is not limited to vocal communication but can also be seen in gestural communication (Heesen et al. 2019; Safryghin et al. 2022).

While adherence to linguistic laws seem widespread in non-human animal vocal communication systems, studying social and ecological factors shaping the phenomenon is of interest to further understand the evolutionary pressures shaping efficiency in animal communication systems.

Marmoset monkeys, *Callithrix jacchus*, can be trained in a vocal conditioning experiment adopt several vocal compression strategies such as increasing call rate, decreasing call duration and increasing the proportion of short calls, which are consistent with the principles of Zipf’s law of brevity (Risueno-Segovia et al. 2023). However, previous studies on common marmoset and the golden-backed uakari, *Cacajao melanocephalus*, fail to find empirical support for Zipf’s law in vocalisations (Bezerra et al. 2011). In rock hyraxes, *Procavia capensis*, Zipf’s law could only be shown in male but not female calls (Demartsev et al. 2019). In different species Menzerath’s Law is only present in certain call-types (tarsiers monkeys, *Tarsius spectrumgurskyae*, titi monkeys, *Plecturocebus cupreus* and gibbons, *Plecturocebus cupreus*: Clink and Lau 2020; Hainan frilled treefrogs, *Kurixalus hainanus*: Deng et al. 2024).

In the present study we investigated adherence to Menzerath’s law in two subspecies of carrion crows, carrion crows, *Corvus corone corone*, hooded crows, *Corvis corone cornix* and hybrids (thereafter crows). Crows belong to the family of corvids, a large family of birds belonging to suborder of oscine passerine birds, with vocal communication playing an important role in the socio-ecology of corvids (Wascher and Reynolds 2025). Corvids mostly produce calls, i.e., short, distinct vocalisations and not song, i.e., heterogeneous, combinatory vocalisations consisting of notes or phrases that are arranged in a specific order and often repeated (Sandoval and Graham 2025). Previously, Menzerath’s law was mostly investigated in song, not calls. Further, Menzerath’s law is mostly investigated on species level and whether it is present in a certain species or not and only recently individual level effects have been described in Java sparrows, *Padda oryzivora* (Lewis et al. 2023). In the present study, we are aiming to investigate how individual factors, such as sex and age as well as social factors like group size, dominance and affiliative relationship status affect adherence to Menzerath’s law. Corvids show high levels of flexibility in their vocalisations, for example common ravens, *Corvus corax*, flexibly adjust calls to audience composition (Szipl et al. 2018) and show similarity in long-distance calls between pair-partners (Luef et al. 2017). Carrion crows have volitional control over calls (Brecht et al. 2019) and can flexibly produce a variable number of vocalisations, with the acoustic features of the first vocalisation being predictive of the total number of calls in a sequence (Liao et al. 2024). Corvids are open-end vocal learners, which means they can acquire new vocalizations throughout their lifetime and not only in a specific sensitive period (Brenowitz et al. 1997). Many corvid species are mimics, allowing them to copy sounds from the environment or conspecifics (Wascher et al. 2025). As vocal communication plays an important role in allowing corvids to navigate social and ecological challenges, we expect widespread rules of signalling efficiency to apply to their calls. We focus on the Menzerath-Altmann law—a precise and more robust mathematical form of Menzerath’s law (Altmann 1980; Torre et al. 2021). Data was collected from carrion and hooded crows as well as hybrids, although we mostly focus on carrion crows as sample size for hooded crows and hybrids is very small (two individuals each).

In carrion crows, we further investigate whether within the species adherence to Menzerath’s law varies, depending on individual factors, such as sex and age and social factors (group size, dominance status and strength of affiliative relationships). We expect weaker adherence to Menzerath’s law in younger individuals compared to adults, as they are expected to still be learning vocalisations patterns. As vocal communication is important for both male and female crows, we do not have specific predictions regarding a sex-based difference how strongly Menzerath’s law is observed in carrion crows. Group size is often considered a driving factor of ‘social complexity’ and associated with this ‘vocal complexity’ (Freeberg and Krams 2015; Krams et al. 2012; Manser et al. 2014). We do expect a higher need for efficiency in larger groups to allow for coordinated vocalisations and behaviour, hence adherence to Menzerath’s law is expected to be higher in larger groups. Lastly, we also investigate effects of dominance and strength of affiliative relationships. We expect the ability to communicate efficiently and hence adherence to Menzerath’s law to be positively correlated with dominance status and strength of affiliative relationships in the group.

## Methods

### Ethical note

The current study was conducted in two populations of captive crows, housed in large outdoor enclosures in Navafría, Castilla y León, Spain (42°36’33 N 5°26’56) and Grünau, Upper Austria (47°51’02 N 13°57’44 E). The crows were kept in captivity before, throughout, and after the duration of the study. Aviary sizes varied between populations and groups from 20 to 72 m^2^ and are always equipped with wooden perches, natural vegetation and rocks. An enriched diet consisting of fruit, vegetables, bread, meat and milk products is provided daily. In both locations water is available *ad libitum* for both drinking and bathing. Keeping of captive birds in Spain was authorized by Junta de Castilla y León (núcleo zoologicó 005074) and in Austria under a licence issued to the Cumberland game park Grünau (AT00009917).

### Study subjects

Data for the present study has been collected from 2010 to 2015. As data for the present study has been recorded over an extended period time, group composition and study subjects changed in different phases, which is summed up in Table 1. Most study subjects are carrion crows, however in the Austrian population there were also two hooded crows and two hybrids (Table 1). Since a recent taxonomic update, carrion crows and hooded crows are considered subspecies of the same species (AviList Core Team 2025). Birds were kept in different group compositions, which reflect natural conditions in the wild: (A) ‘flock’ or ‘family’: groups of three and more individuals, mostly juvenile individuals grouped together but in one case a pair successfully reproducing; (B) ‘pair’: Adult individuals are mostly kept in male-female pairs; in some cases also in trios and due to death of partner for a limited amount of time as singles. All birds were hatched in the wild and most were hand-raised and brought into captivity before fledging, except Valencia and Xufa, who hatched in captivity. Groups were visually but not acoustically separated from each other. For more information on study subjects see Wascher (2021) and Wascher et al. (2019). As an estimate for strength of affiliative relationships for each subject we calculated a Composite Sociality Index (CSI) and as an estimate for dominance rank, we calculated an Elo-rating (ELO) for each subject. We recorded a total of 2,079 individual focal observations (899 in the Austrian population; 1,180 in the Spanish population) lasting five minutes each. All occurring behaviours were recorded but for this study, we focused on the frequencies of agonistic behaviour (threat, chase flight and fight) and affiliative behaviours (allopreen and contact sit). We recorded the identity, role (initiator/receiver) of interacting individuals and the outcome of the agonistic interaction (winner/loser), with the loser of an agonistic interaction defined as the individual that retreated. CSI was calculated according to (Silk et al. 2010). Following Archie et al. (2014), we corrected for a different observational effort between individuals by regressing CSI against the number of observations per individual. We calculated a mean CSI for each individual across all possible dyads, representing the average strength of affiliative relationships. The relative success levels of individuals in agonistic encounters was calculated as an Elo-rating in the R package ‘aniDom’ (version 0.1.4; Sánchez-Tójar et al. 2018). Each individual was rated based on the outcome of each discrete interaction (winner/loser) and the (predicted) probability of that outcome occurring (Neumann et al. 2011). Similar to above, we corrected for a different observational effort between individuals by regressing Elo-rating against the number of observations per individual. Details of behavioural data collection and calculation of CSI and Elo-rating are described in Wascher (2021) and Wascher et al. (2019).

### Recording vocalisations and data analysis

Vocalisations have been recorded during regular behavioural observations using either a Sennheiser ME67 directional microphone on a K6 module, and a 13 Marantz PMD661 digital recorder or a SM1 Wildlife acoustics SongMeter. Recordings were opportunistically taken when an observer was present in the enclosure and hence recording durations differ between individuals (Table 1). Crows were generally habituated to the human observer. The human observer identified the calling individual by speaking on the recording.

**Table 1:** Study subjects. Year of hatching, sex, species, population and group compositions of all individuals contributing to the present study. Dates refer to the times recordings were made in a specific group composition and the number of calls and number of sequences recorded

Individual	Age	Sex	ELOrating	CSI	Subspecies	Population	Group	Dates	# Calls	# Sequences
Alicante	2010	F	160.476	0.865	Carrion	Spain	HL4	2013-01-06 to 2013-11-15	126	50
Baerchen	2008	M	-254.001	3.764	Carrion	Austria	Baerchen, Peter	2010-04-11 to 2015-07-04	1214	399
Bluepoint	2012	M	-109.88	3.093	Carrion	Spain	VR	2013-01-11 to 2013-09-25	68	22
Bluestripes	2012	M	324.532	2.431	Carrion	Spain	VR	2013-01-12 to 2013-06-16	29	11
Cabezon	2010	M	63.983	2.415	Carrion	Spain	VL	2012-12-27 to 2014-04-19	524	175
Franz	2007	M	-582.255	3.218	Carrion	Austria	Franz, Toeffel	2010-04-22 to 2012-07-26	162	53
Gabi	2007	F	116.241	0.477	Carrion	Austria	Gabi, Klaus, Hugo, Resa	2010-04-09 to 2010-09-10	341	118
							Gabi, Klaus, Resa	2011-09-10 to 2012-09-10	315	88
							Gabi	2012-11-10 to 2012-11-15	168	58
Gertrude	2011	F	-216.389	2.181	Hooded	Austria	Gertrude, Walter	02.10.2011-01.06.2013	54	17
GreenO	2011	M	-355.748	0.636	Carrion	Spain	HL4	2012-12-30 to 2013-04-26	191	56
							HL3	2013-05-04 to 2013-09-24	519	148
							GreenO, Whitestripes	2013-11-12 to 2014-04-15	63	20
GreenZ	2011	M	-1.455	0.706	Carrion	Spain	HL4	2013-01-06 to 2014-04-19	104	37
							HL3	2013-05-11 to 2013-11-15	260	89
							GreenZ, White	2014-01-31 to 2014-04-19	251	72
Hugo	Unknown^*^	M	-156.827	4.486	Carrion	Austria	Gabi, Klaus, Hugo, Resa	2010-04-09 to 2010-09-08	59	15
Ibiza	2010	F	-53.24	1.654	Carrion	Spain	HR	2012-12-31 to 2013-05-09	951	293
							HR4	2013-07-01 to 2014-12-25	837	214
Klaus	2009	M	371.871	0.368	Carrion	Austria	Gabi, Klaus, Resa	2011-09-07 to 2012-01-10	290	89
							Gabi, Klaus	2012-01-14 to 2012-09-21	128	44
Martinez	2010	M	46.029	3.29	Carrion	Spain	HR	2012-12-31 to 2013-06-16	320	98
							HR4	2013-06-25 to 2014-12-25	534	150
Munin	2014	F	-139.34	0.107	Hybrid	Austria	Munin, Momo, Willi	2015-05-28 to 2015-05-30	6	3
Nino	Unknown^*^	M	0	0	Hooded	Austria	Nino	2012-07-29 to 2012-07-29	50	19
Olaf	2005	F	0	0	Hybrid	Austria	Olaf, Ruediger	2010-04-12 to 2010-05-01	230	86
Peter	2007	F	-266.528	4.636	Carrion	Austria	Baerchen, Peter	2010-05-13 to 2015-07-01	32	11
Pobla	2010	F	-108	3.706	Carrion	Spain	VL	2013-01-15 to 2014-04-19	1119	332
Redcross	2012	F	-166.673	2.63	Carrion	Spain	VR	2013-01-11 to 2013-09-15	58	18
Redpoint	2012	M	77.096	3.331	Carrion	Spain	VR	2013-01-06 to 2013-09-25	628	157
Redstripes	2012	M	43.234	2.431	Carrion	Spain	VR	2013-01-06 to 2013-01-06	2	1
Resa	2009	F	80.771	0	Carrion	Austria	Gabi, Klaus, Hugo, Resa	2012-01-14 to 2012-07-17	6	2
Ruediger	Unknown^*^	M	0	0	Carrion	Austria	Olaf, Ruediger	2010-04-28 to 2012-07-12	30	6
Toeffel	2008	F	-72.342	9.957	Carrion	Austria	Franz, Toeffel	2010-04-22 to 2012-11-11	648	204
Valencia	2013	M	23.732	0.406	Carrion	Spain	HR4	2013-06-25 to 2014-12-25	588	159
Walter	2011	M	216.389	1.144	Carrion	Austria	Gertrude, Walter	2011-09-07 to 2013-11-27	369	126
							Baerbel, Walter	2015-05-27 to 2015-07-05	77	20
White	2008	F	241.758	1.189	Carrion	Spain	HL4	2012-12-30 to 2013-03-28	379	119
							HL3	2013-05-04 to 2013-11-15	267	101
							GreenZ, White	2014-01-31 to 2014-04-16	18	5
Whitecross	2012	M	-46.987	2.344	Carrion	Spain	VR	2013-01-23 to 2013-09-21	20	7
							Wild	2014-04-12 to 2014-04-18	28	12
Whitepoint	2012	M	58.623	1.042	Carrion	Spain	VR	2013-09-10 to 2013-09-21	7	3
Whitestripes	2012	F	-156.653	0.897	Carrion	Spain	VR	2013-01-11 to 2014-04-15	6	3
Willi	2012	M	139.34	0.107	Carrion	Austria	Munin, Momo, Willi	2015-07-04 to 2015-07-04	6	2
							Toeffel, Willi	2013-11-26 to 2013-11-27	30	11
Xufa	2013	F	-102.11	1.08	Carrion	Spain	HR4	2013-06-25 to 2013-06-25	11	3

^*^For individuals with unknown age, we have used the mean ages of all the other birds

Onsets and offsets of individual calls have been annotated by CAFW using Audacity^®^ or Raven Pro (K. Lisa Yang Center for Conservation Bioacoustics at the Cornell Lab of Ornithology 2024). Vocalizations from unknown callers, overlapping vocalizations, incomplete sequences, and single call sequences have been excluded for the present study. Call sequences can be intuitively recognized by human observers, however for the present study we have classified calls with more than one second inter-call duration as separate sequences. This was manually validated by CAFW. In total, we recorded 12,123 calls in 3,726 complete sequences. For details about calls and sequences per individual, see Table 1.

### Statistical analysis

All models were fit using the lme4 (v1.1-35.1) (Bates et al. 2015) package in R (v4.3.1) (R Core Team 2019). To avoid the many problems associated with p-values, we report mean estimates and 95% Wald confidence intervals and interpret intervals that do not overlap zero as indicating a strong effect. All reported models were manually checked for convergence.

We focus on the Menzerath-Altmann law—a precise and more robust mathematical form of Menzerath’s law (Altmann 1980; Torre et al. 2021). Here is the standard form of the Menzerath-Altmann law where y is the duration of elements within a sequence composed of x elements, and a, b, and c are parameters controlling the shape of the relationship.

1 $$y\, = \,a{x^b}{e^{cx}}$$

If we set c to 0 and apply some simple algebra, we get a linear model that is the most common form of the law in contemporary linguistics (Hou et al. 2017).

2 $$ln\left( y \right)\, = \,ln\left( a \right)\, + \,b*ln\left( x \right)$$

We follow other studies of the Menzerath-Altmann law in nonhuman animals and use the above linear model (Clink and Lau 2020; Favaro et al. 2020; Gustison et al. 2016, p. 201; James et al. 2021; Lewis et al. 2023; Stepanov et al. 2023; Vradi 2021; Youngblood 2024, 2025). y is usually the mean duration of elements within sequences, but we will use the full distribution of call durations within sequences (Youngblood 2024, 2025) to avoid spurious “regression to the mean” effects (Ferrer-i‐Cancho et al. 2013; Gustison et al. 2016; Milička 2023) and better capture uncertainty in the models (Youngblood 2024). We also follow other work in excluding single-element sequences (i.e., with a length of one) from the analysis, which have been shown to depart from Menzerath’s law (Torre et al. 2019, 2021; Heesen et al. 2019; Hernández-Fernández et al. 2019).

The “simple” model, used to assess Menzerath’s law separately in carrion crows, hooded crows, and hybrids of carrion and hooded crows, had the following specification:

3 $$\begin{gathered}ln\left( {call\_duration} \right) \sim \hfill \\\,ln\left( {sequence\_length} \right) + {\mkern 1mu} \left( {1|individual/sequence} \right) \hfill \\ \end{gathered} $$

Nested varying intercepts for sequences and individuals are included to account for the repeated measurement of call durations within sequences and for individual variation in call durations. It is also worth noting that we applied the simple model separately to each species, rather than analysing them together with species as a fixed effect to assess species differences. In this case, given the dramatic disparity in sample sizes between the three species, we think it is unwise to make conclusions about the relative strength of Menzerath’s law, and focus on the presence of it instead.

The “complex” model, used to assess individual variation in Menzerath’s law based on demographic factors, had three potential specifications:

4 $$\begin{gathered} ln\left( {call\_duration} \right){\mkern 1mu} \sim {\mkern 1mu} \hfill \\ ln\left( {sequence\_length} \right){\mkern 1mu} \hfill \\+ {\mkern 1mu} sex{\mkern 1mu} + {\mkern 1mu} group\_size{\mkern 1mu} + {\mkern 1mu} age{\mkern 1mu} + {\mkern 1mu} elo{\mkern 1mu} + {\mkern 1mu} csi{\mkern 1mu} + \, \hfill \\{\mkern 1mu} \left( {1|population/group/individual/sequence} \right) \hfill \\ \end{gathered} $$

5 $$\begin{gathered} ln\left( {call\_duration} \right){\mkern 1mu} \sim {\mkern 1mu} \hfill \\ ln\left( {sequence\_length} \right){\mkern 1mu} \hfill \\*\left( {sex{\mkern 1mu} + {\mkern 1mu} group\_size{\mkern 1mu} + {\mkern 1mu} age{\mkern 1mu} + {\mkern 1mu} elo{\mkern 1mu} + {\mkern 1mu} csi} \right){\mkern 1mu} {\mkern 1mu} \hfill \\+ {\mkern 1mu} \left( {1|population/group/individual/sequence\_id} \right) \hfill \\ \end{gathered} $$

6 $$\begin{gathered} ln\left( {call\_duration} \right){\mkern 1mu} \sim {\mkern 1mu} \hfill \\ ln\left( {sequence\_length} \right) \hfill \\*\left( {sex{\mkern 1mu} + {\mkern 1mu} group\_size{\mkern 1mu} + {\mkern 1mu} age{\mkern 1mu} + {\mkern 1mu} elo{\mkern 1mu} + {\mkern 1mu} csi} \right){\mkern 1mu} \hfill \\+ {\mkern 1mu} \left( {1|population/group/individual/sequence\_id} \right){\mkern 1mu} \hfill \\+ {\mkern 1mu} \left( {0{\mkern 1mu} + {\mkern 1mu} ln\left( {length} \right)|population:group:individual} \right) \hfill \\ \end{gathered} $$

The first of these models includes fixed effects for sequence length, sex, group size, age, ELO, and CSI, as well as nested varying intercepts for population, group, individual, and sequence. The second of these adds interactions between the fixed effect for sequence length and sex, group size, age, ELO, and CSI. The third of these adds additional nested varying slopes that allow for the strength of the effect of sequence length on call duration to vary by population, group, and individual. All three of these models were fit to the data, and the one with the lowest AIC is reported in the results.

Importantly, the complex models were only fit to the data from carrion crows. As seen in Table 1, the data from hooded and hybrid crows are too sparse to assess the effect of demographic factors on Menzerath’s law. For example, both hooded crows come from the Austria population, and both hybrid crows are female, making it impossible to disentangle those factors from species differences. Analysis code and data are available at https://masonyoungblood.github.io/crow_efficiency/.

## Results

**Fig. 1:**
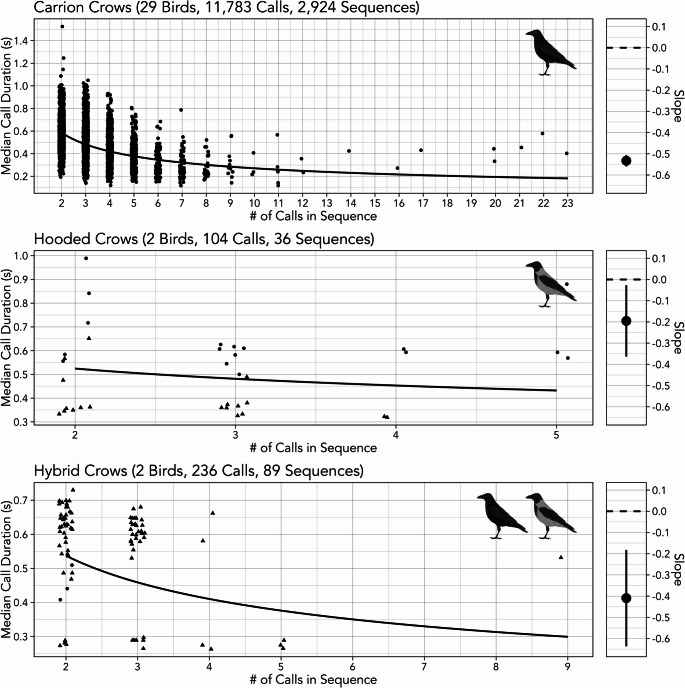
The distribution of median call durations and call sequence lengths (left) and the slope of Menzerath’s law (right) for carrion crows (top), hooded crows (middle), and hybrids of carrion and hooded crows (bottom). Different individuals in hooded crows and hybrid crows are plotted in different symbols. The bars in the slope plot (right) mark the 95% confidence intervals around the point estimates

In all three subspecies, there is a strong negative relationship between sequence length and call duration, consistent with Menzerath’s law (Fig. 1; Table 2). As mentioned in the methods, we chose to not formally analyse species differences in Menzerath’s law because of the large disparities in sample size, but it is interesting to note that the strength of the law in hybrid crows is situated between carrion and hooded crows (Fig. 1; Table 2).

**Table 2:** The results of the simple model of Menzerath’s law applied separately to call sequences from carrion crows, hooded crows, and hybrids of carrion and hooded crows. 2.5% and 97.5% mark the bounds of the 95% confidence intervals around the point estimates, where intervals that do not overlap zero (i.e., strong effects) are marked with an asterisk

Species	Parameter	Estimate	2.5% (Lower)	97.5% (Upper)	
Carrion Crows	Intercept	0.018	-0.161	0.197	
	Length	-0.533	-0.559	-0.507	*
Hooded Crows	Intercept	-0.034	-1.609	1.542	
	Length	-0.195	-0.363	-0.027	*
Hybrid Crows	Intercept	-0.218	-0.930	0.494	
	Length	-0.409	-0.637	-0.182	*

**Fig. 2:**
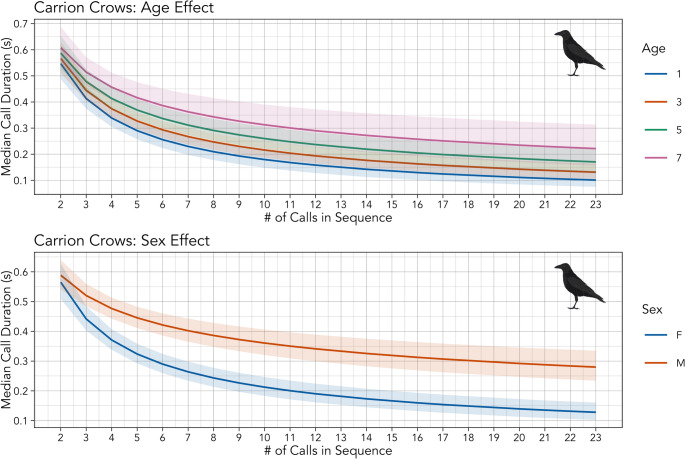
The predicted relationship between element durations and sequence lengths for different levels of age (top) and sex (bottom)

Of the three complex models fit to carrion crow calls, the third—with interactions between demographic factors and the effect of sequence length, and with varying slopes for individual identity—fit the data best (ΔAIC > 2). Likelihood ratio tests indicate Eq. 6 is the best fitting model of individual variation—Eq. 5 outcompetes Eq. 4 (*p* = 4.5 × 10^-32), and Eq. 6 outcompetes Eq. 5 (*p* = 1.8 × 10^-9). The fact that the third complex model (AIC = 14,653) fits better than the second complex model (AIC = 14,698), which excludes varying slopes for individual identity, suggests that there is meaningful individual variation in Menzerath’s law, even after accounting for demographic factors. The results of the third complex model indicate that demographic factors influence both call duration and the strength of Menzerath’s law (Table 3). Call durations are longer in males, older birds, and more dominant birds, and Menzerath’s law appears to be weaker in both males and older birds (Fig. 2).

**Table 3:** The results of the complex model of Menzerath’s law, built to assess individual variation based on demographic factors, applied only to call sequences from carrion crows. 2.5% and 97.5% mark the bounds of the 95% confidence intervals around the point estimates, where intervals that do not overlap zero (i.e., strong effects) are marked with an asterisk. Interactions between demographic factors and Menzerath’s law are marked with colons (e.g., “length: age” is the interaction between age and effect of sequence length on call duration)

Parameter	Estimate	2.5% (Lower)	97.5% (Upper)	
Intercept	-0.267	-0.494	-0.040	*
Length	-0.678	-0.796	-0.560	*
Sex (M vs. F)	0.541	0.254	0.827	*
Group Size	-0.107	-0.214	0.001	
Age	0.142	0.086	0.198	*
ELO	0.201	0.078	0.324	*
CSI	0.081	-0.087	0.250	
Length: Sex (M vs. F)	0.338	0.183	0.494	*
Length: Group Size	-0.043	-0.108	0.022	
Length: Age	0.066	0.014	0.119	*
Length: ELO	0.045	-0.019	0.109	
Length: CSI	-0.033	-0.114	0.048	

## Discussion

In the present study we show that the call sequences of crows—specifically carrion crows, hooded crows, and hybrids of carrion and hooded crows—adhere to Menzerath’s law. In other words, call sequences with more calls are composed of shorter calls, which is suggested to lead to greater communicative efficiency in human language (Piantadosi et al. 2011). To our knowledge, this is the first time efficiency laws have been shown in vocal communication of corvids, adding to the widespread evidence for Menzerath’s law in both human and non-human communication systems (Deng et al. 2024; Gustison et al. 2016; Semple et al. 2010; Youngblood 2024, 2024, 2025). Interestingly, within carrion crows, adherence to Menzerath’s Law was modulated by sex and age. Males’ calls are longer, and their adherence to Menzerath’s law is weaker, which may reflect sex-specific roles in social organization or differences in vocal usage patterns. Linguistic laws previously mostly have been investigated in song, which in many species is sexually selected in males to attract females (Clink and Lau 2020; James et al. 2021; Valente et al. 2021; Youngblood 2024, 2025). Calls in corvids often function as contact calls (Anjos and Vielliard 1993; Berger and Ligon 1977; McCaig et al. 2015; Roskaft and Espmark 1982; Sitasuwan and Thaler 1985), food calls (Bugnyar et al. 2001; Mates et al. 2015; Roehmholdt 2019; Roskaft and Espmark 1982; Szipl and Bugnyar 2014), territory calls (Mates et al. 2015; Sabol et al. 2022; Tanimoto et al. 2017), or alert signals (Chamberlain and Cornwell 1971; Conner 1985; Cornell et al. 2012; Ellis 2009; Mates et al. 2015; Pendergraft and Marzluff 2019; Tanimoto et al. 2017; Yorzinski and Vehrencamp 2009) and are typically emitted by both sexes. In rooks, males produced call units with lower diversity and gradation than females and individual males produced different call repertoires, whereas females produced more similar call repertoires to each other (Martin et al. 2024). Our study indicates that female carrion crows might communicate more efficiently compared to males. This is different to a previous study in lemurs, *Indri indri*, which showed no sex difference in adherence to Menzerath’s law (Valente et al. 2021).

Surprisingly, younger individuals displayed stronger adherence to Menzerath’s Law compared to older crows. This finding contrasts with our original prediction, that efficiency in vocal production would increase with age. This finding contrasts previous findings in zebra finches, *Taeniopygia castanotis*, were song elements and gaps shorten over development (Glaze and Troyer 2013) and humans, were vocal efficiency increases with age (Tang and Stathopoulos 1995). Songbirds reared without auditory experiences that guide vocal development still adhere to Menzerath’s law, indicating that vocal efficiency is linked to vocal production mechanisms rather than learned behaviour (James et al. 2021). Our results could indicate that carrion crow vocalisations are limited to vocal efficiency at younger age and become more flexible with increased age. Since call duration overall also increases with age in our analysis, we suggest the need for informativeness of calls may outweigh the importance of efficiency as crows age. Further studies on the ontogeny of adherence to Menzerath’s law are important.

Group size, dominance status, and the strength of affiliative relationships did not significantly influence adherence to Menzerath’s Law, although call duration itself was longer in more dominant birds. This suggests that, in carrion crows, the structural efficiency of vocal sequences may be relatively stable across different social contexts. However, it should be noted that our data stems from captive individuals, where social contexts such as group composition were under human control, hence the full range of social contexts might not be captured in the data. Overall, our findings highlight the presence of Menzerath’s Law in carrion crow communication and point to individual-level factors, rather than broader group dynamics, as influencing the degree of adherence. Future research should explore whether these patterns hold across different communicative contexts such as different call-types and in other corvid species, to better understand the interplay between social structure, cognitive demands, and communicative efficiency.

## Electronic supplementary material

Below is the link to the electronic supplementary material.

Supplementary Material 1

## Data Availability

Analysis code and data are available at https://masonyoungblood.github.io/crow_efficiency/.
