# Correction: Vocal efficiency in crows

**DOI:** 10.1007/s10071-025-02001-9

**Published:** 2025-08-20

**Authors:** Claudia A. F. Wascher, Mason Youngblood

**Affiliations:** 1 Behavioural Ecology Research Group, School of Life Sciences, Anglia Ruskin University, Cambridge, UK; 2 Institute for Advanced Computational Science, Stony Brook University, Stony Brook, NY USA

**Correction: Animal Cognition (2025) 28:75**

10.1007/s10071-025-01985-8.

In the sentence beginning ‘‘Interestingly, adherence to Menzerath’s law was stronger in males’ in this article, the word ‘males’ should have read ‘females’.

The original article has been corrected.

